# The Role of the Gut Microbiome in Energy Balance With a Focus on the Gut-Adipose Tissue Axis

**DOI:** 10.3389/fgene.2020.00297

**Published:** 2020-04-07

**Authors:** Han Xiao, Sona Kang

**Affiliations:** Department of Nutritional Sciences and Toxicology, University of California, Berkeley, Berkeley, CA, United States

**Keywords:** type 2 diabetes, adipose tissue, microbiome, obesity, metabolism

## Abstract

Obesity is a complex disease attributable to many factors including genetics and environmental influences. Growing evidence suggests that gut microbiota is a major contributing factor to the pathogenesis of obesity and other metabolic disorders. This article reviews the current understanding of the role of gut microbiota in the regulation of energy balance and the development of obesity, and how the microbiota communicates with host tissues, in particular adipose tissue. We discuss several external factors that interfere with the interplay between gut microbiota and host tissue metabolism, including cold exposure, diet regimens, and genetic manipulations. We also review the role of diet-derived metabolites that regulate thermogenesis and thus energy homeostasis. Among the gut microbial metabolites, we emphasize short-chain fatty acids, which could be utilized by the host as a direct energy source while regulating the appetite of the host through the gut-brain axis.

## Introduction

Obesity is a worldwide epidemic, with sedentary lifestyles and increased food intake likely the main causes (Jebb and Moore, 1999; Hu, 2003). However, there are other mechanisms at work. The microbiota in the human gut, also known as gastrointestinal microbiota or gut microflora, describes the microorganisms that live in the digestive tract – the majority of which reside in the large intestine (Thursby and Juge, 2017). Among the tens of trillions of microorganisms present, there are at least 1,000 species of bacteria, consisting of over 3 million genes (Ley et al., 2006; Qin et al., 2010). Remarkably, two thirds of the gut bacterial species are unique to each individual (Lloyd-Price et al., 2016), with many factors influencing the gut microbiota such as hygiene, diet, geographical locations, and host genotype (Chong et al., 2018). This microbial community plays important roles in vital processes such as digestion, vitamin synthesis, and metabolism (Thursby and Juge, 2017). The gut microbiota has gained broad attention in the last decade due to its association with a wide range of diseases, from metabolic disorders, immune diseases, to neurodegenerative diseases and even cancers (O’Keefe, 2016; Liang et al., 2018).

**FIGURE 1:**
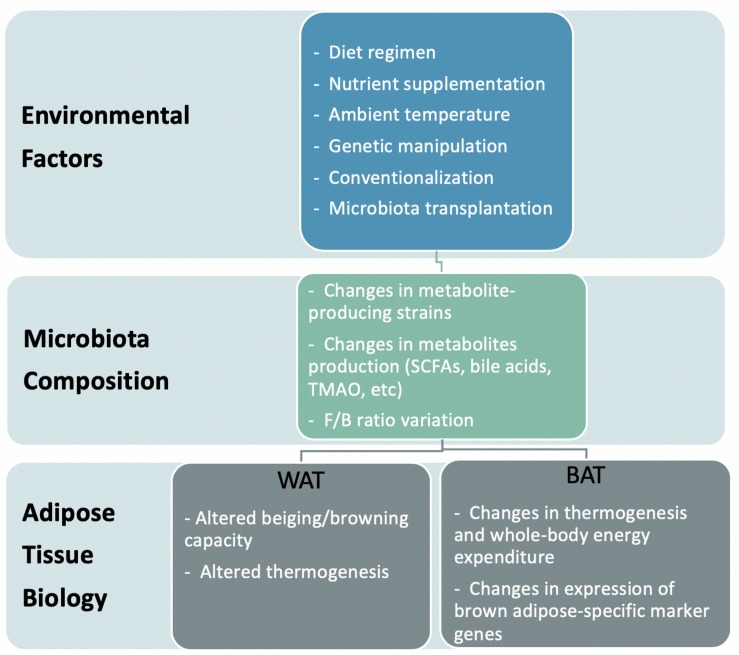
Model representation of the effects of environment-induced altered microbiota on adipose tissue biology.

Recent studies suggest that the composition and activities of gut microbiota not only associate with obesity but cause it. The gut microbiota not only contributes metabolites and energy to the host but also controls the absorption of nutrients in the intestine, thereby influencing human energetics (Thursby and Juge, 2017). Considering the possibility of modulating gut microbiome activity through dietary or biological approaches, the microbiota is an attractive target for medical intervention. This review describes how bacterial metabolites impact both the metabolism and function of adipose tissue, thereby regulating the energy homeostasis of the host. We also discuss the proposed mechanisms of how these bacteria affect the development of obesity (Figure 1).

## Obesity and Gut Microbiota

In recent years, accumulating evidence has shown that gut microbiota influences the pathophysiology of obesity and related metabolic disorders. This started from the observation that conventionally raised mice (i.e., mice raised in contact with microbes) are fatter than germ-free mice (i.e., mice raised in an isolator and without any exposure to microbes) even though they consume fewer calories (Backhed et al., 2004). Then, a mouse study demonstrated that transplanting distal gut communities from genetically obese *ob/ob* mice (Turnbaugh et al., 2006), or diet-induced obese C57BL/6J (Turnbaugh et al., 2008), into germ-free mice is sufficient to recapitulate the obese phenotype in the recipient mouse. Turnbaugh et al. (2009b) conducted a similar study using a humanized mouse model, in which mice received the microbiome of a healthy adult human then underwent different diet regimens – a high-fat diet or a control diet with low fat and abundant polysaccharides. Germ-free mice that received the gut microbiome of humanized mice fed high-fat chow gained significantly more adiposity during the 2 weeks after transplantation than did the recipient mice of the control diet (Turnbaugh et al., 2009b). Similarly, transplanting the gut microbiome of patients after a Roux-en-Y gastric bypass gives rise to reduced fat deposition in germ-free mice (Tremaroli et al., 2015).

To address the causal role of the microbiome in obesity, the microbiomes of human twin pairs discordant for obesity were transplanted into C57BL/6 germ-free mice (Ridaura et al., 2013). These humanized mouse models were fed diets low in fat and rich in polysaccharides (Ridaura et al., 2013). Remarkably, the recipients of the obese microbiota gained more fat than those with the lean microbiota (Ridaura et al., 2013). These differences in body composition were correlated with a different capacity for fermenting nutrients. For instance, lean communities had greater fermentation of short-chain fatty acids (SCFAs), whereas obese communities had increased metabolism of branched-chain amino acids – a difference that has been noted elsewhere to impact the metabolic health the host (Newgard et al., 2009). Interestingly, feeding the mice who received obese microbiota with a diet containing low amounts of saturated fats and high amounts of fruits and vegetables rescued the obese phenotypes (Ridaura et al., 2013). In addition, when obese mice were co-housed with lean mice for 5 days after transplantation, they had less weight gain and a microbiota metabolic profile that leaned toward a lean-like state (Ridaura et al., 2013).

Specific bacteria strains have been associated with obesity and type 2 diabetes. Firmicutes and Bacteroidetes are the two dominant gut microphyla (Qin et al., 2010), and their ratio (the F/B ratio) has been suggested to be correlated with obesity and metabolic health, albeit this remains controversial (Turnbaugh et al., 2006, 2009a). A study on obesity-discordant twins by Turnbaugh et al. (2009a) found that the obese twin had more Firmicutes and more microbiome genes associated with nutrient transporters. The lean twin had a higher relative abundance of Bacteroidetes and more genes linked to carbohydrate metabolism (Turnbaugh et al., 2009a). Shifts in the F/B ratio are also observed across mouse strains with different susceptibilities to diet-induced obesity; the strains of high weight gainers from high-fat feeding have a bigger shift in F/B compared to those with low weight gain (Ley et al., 2005; Turnbaugh et al., 2006). However, other investigations have failed to find significant differences in the F/B ratio between lean and obese humans at both baseline level and after weight loss (Duncan et al., 2008; Zhang et al., 2009; Ismail et al., 2011; Karlsson et al., 2012). Other studies have reported that fecal concentrations of Bacteroidetes are positively correlated with body mass index (BMI) (Ignacio et al., 2016) and that there’s a predominance of Bacteroidetes in obese individuals (Schwiertz et al., 2010). Most likely, these differences are due to different environmental influences including diet, physical activity, and socioeconomic status (Dugas et al., 2016).

Approximately 90% of bacterial species belong to the phyla Firmicutes and Bacteroidetes, with the other important phyla being Actinobacteria, Proteobacteria, and Verrucomicrobia (Jandhyala et al., 2015). While the F/B ratio remains controversial as a determinant of obesity, other bacterial strains have attracted growing interest for their health-promoting effects with less uncertainty, among which *Akkermansia muciniphila* of class Verrucomicrobia has been most extensively studied (Cani and de Vos, 2017). This gram-negative bacterium represents 1–5% of the microbial community and is inversely correlated with obesity-related metabolic disturbances (Schneeberger et al., 2015; Dao et al., 2016). In rodents, treatment with *A. muciniphila* reduces the incidence of obesity and related disorders such as glucose intolerance, insulin resistance, steatosis, and gut permeability (Everard et al., 2013; Plovier et al., 2017). Depommier et al. (2019) found that pasteurized *A. muciniphila* has enhanced beneficial effects on adiposity, glucose tolerance, and insulin resistance when administered to mice (Plovier et al., 2017). The same research team recently confirmed the feasibility of administering *A. muciniphila* to humans by conducting a proof-of-concept translational study in overweight/obese volunteers with insulin resistance (Depommier et al., 2019). They demonstrated the group supplemented with pasteurized *A. muciniphila* had decreased body weight and fat mass, as well as reduced marker levels for liver dysfunction and inflammation. Likewise, a significant association was found between the abundance of *Prevotella* and a human variant linked to a gene related to body fat distribution and insulin sensitivity (Li J. et al., 2018). This area of research deserves additional efforts to understand the interactions of specific bacterial strains with host metabolism, particularly in human subjects.

## Crosstalk Between Gut Microbiota and Adipose Tissue

Adipose tissue is found in two different forms in mammals: white and brown (Peirce et al., 2014). White fat stores energy as triglycerides, whereas brown fat burns extra calories to create heat (Peirce et al., 2014). Additionally, there are beige, or brite, adipocytes, which are an inducible form of brown adipocytes that are sporadically generated within white adipose tissue in response to various stimuli such as cold temperatures (Sidossis and Kajimura, 2015). Accumulating evidence suggests that the gut microbiome affects the thermogenic capacity of brown fat and the formation of beige adipocytes (Suárez-Zamorano et al., 2015; Li et al., 2019). For example, when Suárez-Zamorano et al. (2015) depleted the microbiota of wild-type C57Bl/6J mice by administering antibiotic cocktails, the mice had more beige adipocytes in subcutaneous and perigonadal white adipose tissues (WATs) than did untreated controls. Concordantly, C57Bl/6 germ-free mice in sterile conditions had increased browning (Suárez-Zamorano et al., 2015). Both germ-free and antibiotics-treated mice had more UCP1-positive cells (a marker for thermogenesis) and upregulated brown fat–specific markers in their subcutaneous and visceral WATs (Suárez-Zamorano et al., 2015). Notably, recolonization mostly reverted the metabolic benefits induced by antibiotics treatment. Mechanistically, the authors proposed that increased browning in microbe-depleted mice is due to anti-inflammatory or alternatively activated M2 macrophages derived from upregulation of type 2 cytokines, such as Interleukin 4 (IL 4), Interleukin 13 (IL 13), and Interleukin 5 (IL 5), which promote beige adipogenesis (Martinez et al., 2009; Ganeshan and Chawla, 2014; Qiu et al., 2014; Lee et al., 2015).

However, Li et al. (2019) reported the opposite results. They depleted the microbiota of C57BL/6 mice using a slightly different mix of antibiotics, and unexpectedly, the mice had impaired cold tolerance when exposed to 4°C, accompanied by an impaired ability to induce *Ucp1* expression in BAT and subcutaneous WAT (Li et al., 2019). Consistent with impaired thermogenic gene regulation, the antibiotics-treated mice had reduced whole-body energy expenditure (Li et al., 2019). Unlike the other study, no significant difference was observed regarding M2 macrophage biology (Li et al., 2019). The authors proposed that, instead of macrophage polarity, the gut microbiota influences BAT metabolism by generating certain metabolites (i.e., butyrate) (Li et al., 2019).

The contradictory results between the two studies could arise for multiple reasons. First, despite the identical genetic background, gender, and age of the mice, they were raised in different facilities, meaning they were under unique environmental conditions that may have differentially impacted the microbiota composition before and after antibiotics. In fact, Li et al. (2019) were not able to recapitulate the previous findings, even when they treated the germ-free mice with the same cocktail of antibiotics. However, the mice were treated for different lengths of time. Mice were treated for 6 weeks in the first study (Suárez-Zamorano et al., 2015) and 30 days in the latter (Li et al., 2019), which could have resulted in varying degrees of perturbing the gut microbiota. It is possible that incomplete deletion of microbiota or imbalanced microbiota composition could differentially impact host organs. Nevertheless, both studies suggest that drastic perturbations of gut microbiota have profound impacts on BAT function or the browning process.

In addition to adipocyte thermogenesis, the gut microbiome may affect lipid metabolism (Backhed et al., 2004). Conventionalizing germ-free C57BL/6J mice leads to a rapid and significant increase in body fat content and the development of insulin resistance (Backhed et al., 2004). Moreover, colonization with a single saccharolytic bacterial species, *B. thetaiotaomicron*, a prominent member of the human distal gut microbiota with an extraordinary capacity for acquiring and degrading plant polysaccharides (Xu et al., 2003), also produces a significant increase in total body fat content (Backhed et al., 2004). Mechanistically, it was proposed that the increased adiposity in conventionalized germ-free mice is partially due to the microbial suppression of the intestinal expression of angiopoietin-like protein 4 (*Angptl4*), a secreted protein that inhibits lipoprotein lipase activity (Backhed et al., 2004). Notably, reduced adiposity in germ-free mice is associated with an elevated level of ANGPTL4, accompanied by the increased expression of Pparg coactivator 1 alpha (PGC1A) and genes involved in fatty oxidation in muscle (Bäckhed et al., 2007). Together, these results suggest that the microbiome communicates with adipose and other metabolic tissues to influence host lipid metabolism.

## The Upstream Signals That Elicit the Crosstalk Between Microbiota and Adipocytes

The gut microbiome is highly influenced by food intake and various environmental and genetic factors. In this section, we will discuss which external factors affect the gut microbiota community and influence the metabolism of target tissues, namely white and brown adipose depots (Table 1).

**TABLE 1:** A list of the previously identified key environmental factors that lead to gut microbiota-mediated biological changes in adipose tissue.

Environmental factors	Changes in microbiota composition or metabolite	Altered adipose tissue biology
Transplanting the microbiomes of human twin pairs discordant for obesity into C57BL/6 germ-free mice (Ridaura et al., 2013)	• Lean communities had greater fermentation of short-chain fatty acids (SCFAs)	• The recipients of the lean microbiota gained less fat than those with the obese microbiota
Transplanting microbiota from mice exposed to cold to germ-free mice (Chevalier et al., 2015)	• Increased Bacteroidetes/Firmicutes ratio in the donor gut ∙ Shifts in the major bacterial phyla in the donor gut without changing the overall bacterial diversity	• Enhanced browning and thermogenesis ∙ Promoted energy expenditure and cold tolerance ∙ Improved insulin sensitivity
Reducing the ambient temperature (Ziȩtak et al., 2016)	• Increased Firmicutes/Bacteroidetes ratio ∙ Shifts in the microbiome composition at the phylum and family levels in response to cold exposure ∙ Elevated conjugated bile acid levels associated with the upregulated *Cyp7b1* in liver	• Activated TGR5 receptors ∙ Increased browning in white adipose depots ∙ Higher thermogenic gene expression level in interscapular BAT ∙ Increased overall insulin sensitivity in germ-free mice who received microbiota from cold-exposed mice
An every-other-day fasting (EODF) regimen performed in diet-induced obese mice (Li et al., 2017)	• A reduced Firmicutes to Bacteroidetes ratio	• Weight loss and an improved metabolic profile ∙ Increased beige fat development
Adipose-specific deletion of Napepld (Geurts et al., 2015)	• Changes in the abundance of 64 operational taxonomic units	• Increased fat mass and decreased expression of BAT-specific genes in Germ-free mice receiving the KO microbiota
β-Klotho deficiency (Somm et al., 2017)	• Increased production of the secondary bile acid deoxycholic acid, an activator of TGR5 receptor	• The Klb-KO mice exhibited lean phenotypes with enhanced thermogenesis
Gpr43 knockout (Kimura et al., 2013)	• Increased gut population of SCFA-producing phylum Firmicutes, increased fecal and plasma acetate concentrations in Gpr43-KO mice	• The body weights and WAT weights of both NC- and HFD-fed Gpr43 KO mice were significantly higher, accompanied by significantly increased insulin resistance

### Ambient Temperature

A change in ambient temperature is one of the strongest physiological stimuli for increasing thermogenic adipose formation and activity (Cypess et al., 2009; Lee et al., 2014). A recent study by Chevalier et al. (2015) found that a prolonged cold exposure in C57BL6 mice led to a marked change in the composition of gut microbiota, especially increasing the Firmicutes abundance over Bacteroidetes from 18.6% at RT up to 60.5%. Furthermore, transplanting microbiota from a mouse exposed to cold increased browning in white adipose depots and increased insulin sensitivity in recipient mice (Chevalier et al., 2015). Another study reported similar findings, showing that gut microbiota from cold-conditioned mice modulated fat accumulation by promoting thermogenesis (Ziȩtak et al., 2016). Recipient mice colonized with microbiota from donors that were fed with a high-fat diet and kept at 12°C had reduced fat mass as well as significantly higher mRNA and protein expression of thermogenic genes like Mitochondrial uncoupling proteins1 (*Ucp1*) and Type II Iodothyronine Deiodinase (*Dio2*) in their interscapular BAT compared to mice that received microbiota from donors housed at thermoneutrality (29°C) (Ziȩtak et al., 2016). The role of gut microbiota as a mediator of cold-induced thermogenesis was further explored by Bo et al. (2019) using male Brandt’s voles. They verified that cold exposure modified gut microbiota and increased the concentration of SCFAs. In addition, norepinephrine injection also induced a long-term decrease in food intake and body mass in the treated group, paralleled by altered gut microbiota composition. They confirmed that transplantation of cold-acclimated microbiota triggered thermogenesis in the recipient by activating the cAMP–PKA–pCREB signaling pathway. Therefore, gut microbiota may interact with host neurotransmitters to regulate thermogenesis and energy expenditure during cold acclimation.

### Diet Composition and Diet Regimens

A direct link has been established between gut microbiota and diet-induced obesity (Bäckhed et al., 2007). For instance, bacterial lipopolysaccharide, or endotoxin, has long been identified as an inflammatory factor triggering the onset of obesity, insulin resistance, and diabetes (Cani et al., 2007; Harte et al., 2012). Bacterial endotoxin is abundant in the human gut and circulates at low concentrations in the blood of all healthy individuals, whereas an elevated concentration of endotoxin has been demonstrated to affect the function of the major organs involved in maintaining glucose and lipid homeostasis, including adipose tissue (Caesar et al., 2015; Schroeder and Bäckhed, 2016). Obese and diabetic people have increased plasmatic lipopolysaccharide levels (Harte et al., 2012). The increase in the proportion of gram-negative microbiota, increased gut mucosal permeability, and the consumption of high-fat diets increase the plasmatic lipopolysaccharide levels, which will elicit local inflammatory signals that can further deteriorate the gut barrier and promote bacterial translocation. Cani et al. reported that a 4-week high-fat diet chronically increased the proportion of an LPS-containing microbiota as well as the plasma LPS concentration in mice by two to threefold (Cani et al., 2007). The induction of the elevated plasma LPS level, often referred to as metabolic endotoxemia, in mice is followed by the onset of liver insulin resistance and an increased expression of inflammatory markers in the adipose tissue. Another study also confirmed the onset of insulin resistance induced by low-dose LPS infusion in healthy humans, which is exclusively associated with stimulation of inflammatory pathways as opposed to the insulin resistance caused by intravenous fat or glycerol treatment (Nowotny et al., 2012). This demonstrates that metabolic endotoxemia dysregulates the inflammatory profiles, and triggers body weight gain and insulin resistance. These findings suggest to lower plasma LPS concentration could be a strategy for the control of metabolic diseases. Overall, the gut microbiome may be a potential target for improving metabolic homeostasis.

Diet-induced weight-loss intervention improves low gut microbial gene richness, thus mitigating the dysmetabolism of obese patients (Cotillard et al., 2013). Considering that gut microbiota is, to a large extent, modulated by diet in humans, dietary interventions are a potential means to prevent or treat obesity via modifying gut microbiota composition (David et al., 2014). Obesity is associated with reduced brown adipose activity, which is characterized by impaired β-adrenergic signaling, vascular rarefaction, larger lipid droplets, inflammation, and decreased mitochondrial respiration (Orava et al., 2013). Paradoxically, increased UCP1 expression (a marker for brown adipose) is observed after feeding rodents a high-fat diet (Surwit et al., 1998). Yet, whether this induction of UCP1 serves to “buffer” metabolic dysfunction is unknown. High-fat foods alter the microbiome composition to favor the proliferation of gram-negative bacteria strains (Kasselman et al., 2018) that produce SCFAs, which have been proposed to be associated with increased energy expenditure and thermogenesis (Gao et al., 2009). Thus, it is plausible that the microbiome is the mechanism for high fat diet–induced *Ucp1* expression.

In addition, germ-free mice are resistant to high-fat diet-induced obesity, accompanied by malabsorption of dietary lipids, while HFD-fed SPF mice develop obesity and exhibit an increased abundance of the family Clostridiaceae in their intestines (Martinez-Guryn et al., 2018). The fact that treatment with Clostridiaceae directly upregulates lipid transport genes both *in vivo* and *in vitro* indicates that gut microbiota plays a role in shaping host adaptability to dietary lipid uptake, possibly as early as in the digestive phase.

The ketogenic diet consists of high fat, very low carbohydrate, and moderate protein (Dashti et al., 2004; Paoli, 2014) amounts and has become a popular dietary choice for treating epilepsy, obesity, or neurodegenerative disorders (Paoli, 2014). This diet phenocopies several biochemical characteristics of fasting including reduced insulin level, Forkhead box O (FoxO) signaling, and inhibition of the mammalian target of rapamycin (mTor), and activation of AMP-activated protein kinase (AMPK) (Veech et al., 2017). Recent studies suggest the ketogenic diet significantly impacts gut microbiota, but with mixed results. For example, a mouse study noted that the ketogenic diet increased beneficial species of gut microbiota including *A. muciniphila* and *Lactobacillus* bacteria, which are capable of producing small-chain fatty acids (SCFAs) that may provide the host with beneficial health outcomes (Ma et al., 2018). However, these changes occurred at the expense of reduced overall microbial diversity, possibly due to the minimized carbohydrate intake, which can disrupt other beneficial microbes (Swidsinski et al., 2017). Human studies also point out some potential negative effects on the gut microbiota. For example, overweight and obese subjects following the ketogenic diet for 8 weeks had a significantly reduced amount of a beneficial bacterium, *Bifidobacterium*, in their colon and decreased plasma levels of SCFAs (Brinkworth et al., 2009). Future studies are warranted to establish with more accuracy the effects of high fat–containing diet regimens on microbiota composition and diversity and the mechanisms whereby this microbiota affects adipose biology.

Intermittent fasting has also caught mainstream attention for its many benefits including weight loss. For example, an every-other-day fasting (EODF) regimen in diet-induced obese mice results in weight loss and an improved metabolic profile associated with increased beige fat development (Li et al., 2017). Notably, this was accompanied by a reduced F/B ratio. Moreover, the microbiota seems to play a causal role, as mice that received the microbiota from EODF mice recapitulated the beneficial phenotype while microbiota-depleted mice did not (Li et al., 2017). Another dietary pattern that has beneficial effects on host health is the Mediterranean diet. Chronic intake of the Mediterranean diet or a low-fat diet over a year is associated with an improved insulin profile and modified lipid metabolism in obese patients with coronary heart disease, an effect which is linked to an increased abundance of *Roseburia* genus and *F. prausnitzii* in the gut (Haro et al., 2016). Consistently, other studies have shown that obese patients with metabolic dysfunction have reversed microbiota dysbiosis upon long-term consumption of the Mediterranean diet (Haro et al., 2017).

However, short-term dietary interventions appear to have no effect. A randomized 1-week-long dietary intervention found no effects of different types of bread on clinical parameters nor on gut microbial composition (Korem et al., 2017). Another study comparing the effect of consuming a high-cholesterol diet for 12 weeks also detected no major differences in microbial composition between control and treated mice (Dimova et al., 2017). Together, these results suggest that short-term dietary intervention may not overcome interpersonal variability in gut microbiota composition, and understanding dietary effects requires integration of person-specific factors.

### Genetic Manipulation

Several studies have proposed that some of the metabolic phenotypes in certain genetic mouse models are derived from altered gut microbiota. For example, the endocannabinoid system consists of ubiquitous bioactive lipids that regulate glucose and lipid metabolism, food intake, and inflammation through various receptors in autocrine and paracrine manners (Di Marzo and Matias, 2005; Horn et al., 2018). *N*-acylethanolamines (NAEs) are the best characterized endocannabinoids, and increased NAE levels are associated with obesity and metabolic comorbidities (Tsuboi et al., 2018). Unexpectedly, a mutant mouse carrying an adipose-specific deletion of *Napepld*, which encodes *N*-acylphosphatidylethanolamine phospholipase D (NAPE-PLD), the NAE endocannabinoid synthesizing enzyme, is more susceptible to diet-induced obesity and metabolic dysregulation (Geurts et al., 2015). In these mice, the gut microbiota composition changes in the abundance of 64 operational taxonomic units (Geurts et al., 2015). Germ-free mice receiving the KO microbiota have significantly increased fat mass and decreased expression of BAT-specific genes compared to those who received WT microbiota (Geurts et al., 2015). Similarly, another study (Somm et al., 2017) attributed the altered microbiome as the reason behind the surprising metabolic phenotype in the global knock-out of *Klb* that encodes β-Klotho, which is an obligate co-receptor for FGF21, a hepatokine that stimulates thermogenesis, glucose uptake, and lipolysis (Kharitonenkov et al., 2005; Hondares et al., 2011). Thus, *Klb* KO mice were expected to be resistant to the beneficial action of FGF21 treatment. However, the *Klb*-KO mice are leaner and have more brown adipose activity compared to controls on a high-fat diet (Somm et al., 2017). The authors proposed that the surprising body weight phenotype is attributable to the changes in the host bile acid metabolism (Somm et al., 2017), which is discussed later in this review. These examples illustrate that there are intricate interactions among the microbiome, adipose tissue, and genetics.

## Signals That Mediate the Crosstalk Between Microbiota and Host Tissues

It has been proposed that the microbiota employs microbial and host metabolites for microbiome-to-tissue communication to regulate cellular function. The short-chain fatty acids (SCFAs) acetate (C2), propionate (C3), and butyrate (C4), which are the major end products of microbial fermentation of dietary fiber, serve as the primary energy source for colonic epithelium (Boffa et al., 1978; Candido et al., 1978). A randomized clinical study found that deficiency in SCFA production is associated with type 2 diabetes (Zhao et al., 2018). Zhao et al. (2018) observed that a high-fiber diet promoted the growth of specific SCFA-producing strains in diabetic patients. Participants exhibited better improvement in their blood glucose profiles when they had fiber-promoted SCFA producers in higher abundance and greater diversity. This could partially be mediated by increased glucagon-like peptide-1 production. However, it could be challenging to measure the contribution of each subtype of SCFAs during this process accurately (Zhang et al., 2019). Even if their levels can be measured in feces, they may not adequately reflect the total amount processed by microbiota. For instance, much of the butyrate pool is consumed for energy by the cells lining the colon and so will not enter the fecal pool (O’Keefe, 2016). Considering these constraints, Sanna et al. (2019) adopted Mendelian Randomization to establish the causal relationships between SCFAs and obesity and type 2 diabetes. They found that increased gut microbial activity producing butyrate is positively associated with insulin sensitivity, whereas a higher production of propionate is associated with increased risk of type 2 diabetes (Sanna et al., 2019). Therefore, targeted restoration of certain producers of SCFA subtypes may be a promising approach for managing T2DM.

Several studies suggest that SCFAs activate G protein–coupled receptors (GPCR) to affect the target organ of the host. For example, Gpr43, which has a strong affinity to acetate and propionate, plays an important role in mediating the microbial input in WAT metabolism (Brown et al., 2003; Hong et al., 2005). Gpr43-KO mice are obese compared to their wild-type counterparts on both chow and high-fat diet, whereas adipose-specific Grp43-transgenic mice are leaner and more insulin sensitive (Kimura et al., 2013). Remarkably, the body weight phenotype was abolished under germ-free conditions, demonstrating the importance of microbial metabolism in forming ligands for adipose GPR43 signaling (Kimura et al., 2013). Analysis of the gut microbiota communities revealed that Gpr43-KO mice display an increased gut population of Firmicutes (Kimura et al., 2013), which is an SCFA-producing phylum (Macfarlane and Macfarlane, 2003). This was also accompanied by increased fecal and plasma acetate concentrations in the KO mice (Kimura et al., 2013). In support of the role of Gpr43 and acetate, anti-lipolytic activity of GPR43 in WAT was reported (Robertson et al., 2005). Moreover, acetate-dependent GPR43 stimulation in the WAT, but not in muscles or liver, improves glucose and lipid metabolism (Kimura et al., 2013). Together, these findings suggest that acetate-mediated GPR43 signaling in WAT may have metabolically beneficial functions.

A recent study proposed that butyrate mediates the thermogenic stimulation of BAT, as administering butyrate sodium to microbiota-depleted mice partially rescues impaired thermogenesis and promotes fat oxidation (Li et al., 2019). Other studies have revealed the association between butyrate administration and improved blood glucose profiles (Xu et al., 2018), obesity-related lipid accumulation, and low-grade chronic inflammation (Fang et al., 2019). Fang et al. (2019) confirmed that administering sodium butyrate to mice re-shapes their gut microbiota composition to favor an improved intestinal barrier, leading to lower serum lipopolysaccharide concentrations. However, it is worth noting that oral butyrate treatment does not seem to improve human BAT activity when it is administered to lean or metabolic syndrome subjects in spite of a significant improvement the butyrate administration resulted in lean subjects’ insulin sensitivity (Bouter et al., 2018). As the butyrate dose used in this study was lower than the that usually given in mouse studies, a sub-therapeutical dose of sodium butyrate might be an explanation for the less optimal response by the metabolic syndrome subjects. Therefore, larger placebo-controlled trials with different dosage and age-matched subjects are needed to better understand the potential of oral butyrate treatment as an effective means for glucose regulation in human subjects with metabolic syndrome and/or Type 2 Diabetes Mellitus. Though most of the butyrate in the cecum may be used by mitochondria as an energy substrate in the colon (den Besten et al., 2013), some enters the circulation system and crosses the blood-brain barrier via monocarboxylate transporters (MCTs) (Vijay and Morris, 2014). Of note, isotope tracing revealed that gut-produced butyrate is mainly routed to the brain rather than peripheral tissues, suggesting that gut-derived butyrate activates BAT via the gut-brain neural circuit, rather than working on adipose directly (Li et al., 2019). This was further supported by the finding that butyrate administration decreases food intake and inhibits orexigenic neuron activity in the hypothalamus (Frost et al., 2014; Li Z. et al., 2018). In line with this study, high-fat-diet-fed mice that receive dietary butyrate for 12 weeks upregulate the expression of UCP1 and PGC1α and have increased mitochondrial function and biogenesis in BAT (Gao et al., 2009). It is noteworthy that butyrate, and to a lesser degree, propionate, are histone deacetylate (HDAC) inhibitors (Waldecker et al., 2008), and pharmacological and genetic inhibition of class I HDAC in particular, has the same effect. Administration of propionate and butyrate to the stromal vascular fraction of porcine adipose tissue enhanced adipocyte differentiation, which could be partially mediated by their inhibitory effect on histone deacetylase activity (Li et al., 2014). Moreover, Krautkramer et al. (2016) demonstrate that administration of SCFAs to germ-free mice retrieved chromatin modification and transcriptional responses associated with microbial colonization. Thus, it is conceivable that the microbiome may affect the epigenome of target tissues.

Other gut microbial metabolites are involved in epigenetic regulation. Exposure of mouse ileal organoids to SCFAs and products generated by *A. muciniphila* modulates histone deacetylase level and the expression of genes involved in satiety and host lipid metabolism (Lukovac et al., 2014). Furthermore, Virtue et al. (2019) identified the importance of tryptophan-derived metabolite indole-3-carboxylic acid (I3CA) produced by the gut microbiota. HFD upregulates adipocyte *miR-181* expression during obesity, which is a microbiota-dependent process. Through controlling the expression of the miR-181 family in white adipocytes in mice, the gut microbial metabolite I3CA plays a role in regulating the energy expenditure and insulin sensitivity of the host. These results indicate that gut microbiota–derived metabolites could be part of the epigenetic mechanisms that regulate host metabolism and adipocyte function in response to dietary modification. In addition to gut microbiota-derived metabolites, the predominant bacterial phyla in the gut has also been shown to correlate with DNA methylation patterns. Although most of the existing evidence focus on the influence of gut microbiome on the epigenetic regulation of host genes involved in maintaining intestinal homeostasis and regulating the mucosal immune system in the gut (Takahashi et al., 2011; Takahashi, 2014), there are a few reports underlining the association of the gut microbiota with differentially methylated genes linked to metabolic diseases. A whole-genome methylation analysis conducted by Kumar et al. (2014) revealed a clear correlation of blood DNA methylation profiles with gut microbiota patterns in pregnant women. Eight pregnant women were classified into two groups depending on their dominant gut microbiota, i.e., *Bacteroidetes*, *Proteobacteria*, and *Firmicutes*. Next-generation sequencing of DNA methylomes indicated that the genes with differentially methylated promoters in the High *Firm* group was functionally associated with lipid metabolism, obesity, and the inflammatory response. Another genome-wide analysis of DNA methylation also demonstrate that the DNA methylation status is associated with gut microbiota composition in obese subjects (Ramos-Molina et al., 2019). Ramos-Molina et al. (2019) found that in adipose tissue, both *HDAC7* and *IGF2BP2* were hypomethylated and overexpressed in the obese subjects with low Bacteroidetes-to-Firmicutes (BFR) ratio compared with the high BFR obese group. This finding indicates that the expression levels of genes implicated in glucose and energy homeostasis in adipose tissue could be epigenetically regulated by gut bacterial populations. In support of this, Remely et al. (2014) discovered that the promoter region of Free Fatty Acid Receptor 3 (FFAR3) showed a significant lower methylation in obese and type 2 diabetics subjects who had a reduced microbial diversity and abundance of *Faecalibacterium prausnitzii*. Their results disclosed the influence of different composition of gut microbiota in obesity and type 2 diabetes on the epigenetic regulation of genes. On the other hand, some reports have demonstrated that fecal micro-RNAs (miRNAs) can shape the composition of the gut microbiome (Liu et al., 2016), showing a potential role for miRNAs in mediating the host-microbiome interaction. Together, these insights are pivotal pieces of information revealing the associations between gut microbiota composition and epigenetic status contributing to host metabolism regulation.

It is well-established that the bacterial-derived metabolite Trimethylamine N-oxide (TMAO) is strongly associated with cardiovascular risks and host inflammation (Zhu et al., 2016). Choline and L-carnitine are the major precursors of TMAO, and they are highly abundant in the Western diet (Koeth et al., 2013; Chen et al., 2017). Western diets consumption result in the production of TMA in gut microbiota, which is metabolized to trimethylamine-N-oxide (TMAO) by host hepatic enzyme flavin-containing monooxygenase 3 (FMO3) (Koeth et al., 2014; Chen et al., 2017). Of note, TMAO is also upregulated in type 2 diabetes, and is associated with obesity traits (Gao et al., 2014). Schugar et al. (2017) proposed that the TMA/FMO3/TMAO pathway is a microbe-to-host endocrine axis that mediates the crosstalk with adipose tissue, as deleting TMAO-producing *Fmo3* increases browning of gonadal WAT and protects against obesity in mice. Complimentary mouse and human studies indicate a negative regulatory role for FMO3 in the browning of white adipose tissue (Ussar et al., 2014). Since TMA results from nutrients commonly consumed in a high-fat diet and is exclusively generated by certain communities of the gut microbiome, dietary intervention or targeting the specific microbes that generate TMAO may have therapeutic implications.

In addition to SCFAs, bile acids appear to play an important role in mediating the interactions between microbiota and host tissues. Bile acids are synthesized from cholesterol by a process orchestrated by multiple liver enzymes (Russell, 2009). Afterward, the gut microbiota promotes deconjugation, dehydrogenation, and dehydroxylation of primary bile acids in the distal small intestine and colon, thus forming secondary bile acids and affecting bacterial composition (Ridlon et al., 2006; Sayin et al., 2013). For instance, conjugated bile acid has been shown to decrease bacterial overgrowth, bacterial translocation, and endotoxemia in rats (Lorenzo-Zúñiga et al., 2003). Bacterial translocation was less in cirrhotic animals receiving conjugated bile acids, while endotoxemia was also reduced by conjugated bile acid feeding (Lorenzo-Zúñiga et al., 2003). A recent study by Ziȩtak et al. (2016) found that cold exposure activates an alternative bile acid synthesis pathway by increasing the expression of Cytochrome P450 Family 7 Subfamily B Member 1 (*Cyp7b1*), a hepatic enzyme that mediates alternative bile acid synthesis. The elevated bile acid secretion changed the microbiota composition and increased adipocyte thermogenesis (Ziȩtak et al., 2016). Confirming this, Worthmann et al. (2017) reported that cold exposure in mice triggers the hepatic conversion of cholesterol to bile acids via the alternative synthesis pathway. At the same time, cold results in accelerated fecal excretion of bile acids via increased CYP7B1. This process is accompanied by alterations in gut microbiota and promotes thermogenesis (Worthmann et al., 2017). In line with these results, it was proposed that the lean phenotype of the Klb-KO mice is due to increased production of the secondary bile acid deoxycholic acid, which is produced by microbiota from hepatic cholic acid (Somm et al., 2017). Notably, deoxycholic acid signals through a G-protein coupled bile acid receptor TGR5 in the BAT to enhance thermogenesis (Somm et al., 2017). Furthermore, KO mice treated with vancomycin, which preferentially targets gram-positive bacteria, including the *Clostridium* species, which is classically described as being responsible for the conversion of primary bile acids into secondary bile acids, reversed the metabolic phenotypes in the KO mice (Somm et al., 2017). This result supports the role of bile acids as mediators of microbiota-mediated thermogenesis. Therefore, interventions targeting the synthesis and/or excretion of bile acids could alter gut bacterial composition, thereby modulating host energy expenditure.

Miyamoto et al. (2019) proposed that gut microbiota confers host resistance to HFD-induced obesity by modulating dietary PUFAs metabolism. Supplementing the diet with 10-hydroxy-cis-12-octadecenoic acid (HYA), a dietary linoleic acid–derived gut-microbial metabolite whose level is significantly reduced by HFD feeding, attenuates various aspects of HFD-induced obesity in mice, including their appetite, body weight, WAT adipocyte size, and blood glucose and insulin level, by promoting GLP-1 secretion via GPCRs. Furthermore, HYA treatment does not elicit adipose inflammation, as opposed to regular linoleic acid supplementation, which is known to be involved in mediating inflammatory responses via the arachidonic acid cascade. Moreover, Lactobacillus-colonized mice show similar effects with elevated HYA levels. The findings illustrate the interplay between gut microbiota and host energy metabolism via the metabolites of dietary omega-6-FAs, thereby shedding light on the prevention and treatment of metabolic disorders by targeting gut microbial metabolites. Another metabolite of linoleic acid that is involved in the regulation of host energy metabolism is 10-oxo-12(Z)-octadecenoic acid (Goto et al., 2015; Kim et al., 2017). It is produced by lactic acid bacteria in the intestine. This production has been linked to activation of adipogenesis (Goto et al., 2015), the mitigation of obesity-related metabolic dysfunction (Goto et al., 2015; Kim et al., 2017), as well as the upregulation of UCP1 expression in WAT via the activation of transient receptor potential vanilloid 1 (Kim et al., 2017).

Another nutrient whose gut microbial metabolites have been associated with adipocyte function is resveratrol. Resveratrol is a non-flavonoid polyphenol compound that is naturally found in a wide variety of plants, such as grapes and peanut skin (Liao et al., 2018). Besides its multiple benefits to host health, such as antioxidation and anti-inflammation, resveratrol potentially alters the composition of gut microbiota to ameliorate adiposity and improve glucose homeostasis in HFD-fed mice (Qiao et al., 2014; Sung et al., 2017). Wang et al. (2020) confirmed the effect of resveratrol-induced gut microbiota by transplanting the microbiota from donors treated with a resveratrol diet for 16 weeks to the HFD-fed mice. They found that the recipient mice showed decreased body weight and improved insulin resistance. In addition, resveratrol-microbiota could modulate lipid metabolism and induce WAT browning in the high-fat diet-fed recipient. Similarly, Liao et al. (2018) found that resveratrol treatment significantly alleviates gut microbiota dysbiosis in HFD-fed mice while promoting WAT browning. This observation is also consistent with previous findings of the promoting effect of resveratrol on brown and beige adipocyte development (Wang et al., 2015; Bird et al., 2017; Zou et al., 2017). Furthermore, similar effects have also been reported for other polyphenol-rich dietary compounds, like cranberry extract (Anhê et al., 2015; NCT03754504,2018). Likewise, gut-produced vanillic acid, the metabolite of another antioxidant, anthocyanins, has also been shown to activate thermogenesis and promote browning in HFD-fed mice (Han et al., 2018).

## Concluding Remarks

The evidence strongly suggests that alterations in gut microbiota diversity and composition contribute to the pathogenesis of obesity and obesity-related metabolic disorders. However, the limitations and pitfalls of the studies should be noted. Many of these studies are observational. In addition, differences in the lab environment could contribute to differences in the microbiome and its effects. Also, many studies used mice, which have gut anatomies that are different from humans. Future studies are needed to gain a more accurate understanding of host-microbiome communications. Based on the existing preliminary data identifying epigenetic mechanisms as a regulator of gut microbiota composition, a dietary approach targeted to favor a more beneficial bacterial population and thus epigenetic changes might be effective in the prevention of obesity. For this, the accurate analysis of microbiome diversity and composition will be key. Large-scale sequencing studies have already identified organisms and their relative abundance in purified DNA by sequencing specific regions of the 16S or 18S ribosomal genes (Qin et al., 2010; Tkacz et al., 2018). In parallel, comprehensive biochemical and metabolomic profiling approaches will be needed to understand the mechanistic basis of host-microbiome interactions.

Accumulating studies have started to examine the effect of gut microbiota transplantation on insulin sensitivity and microbiota composition in humans. Vrieze et al. (2012) reported that infusing microbiota from lean donors to obese male recipients with metabolic syndrome significantly increased their insulin sensitivity. Also, case reports have suggested that intestinal bacteria upon fecal microbiotiota transplantation might affect bodyweight and insulin sensitivity of the recipient (Kootte et al., 2017; De Groot et al., 2019). The duration of thereapeutic effect of FMT is not clear yet; Till then, dietary intake is probably the easiest way to influence intestinal microbiome composition and may even restore pathological disturbances. Considering the causal role of the gut microbiome in human obesity and its manageability through dietary or biological approaches, targeting the microbiome may present a new avenue for therapeutic interventions for preventing and treating obesity and its related metabolic disorders.

## Author Contributions

SK and HX co-drafted manuscript. HX crafted art work.

## Conflict of Interest

The authors declare that the research was conducted in the absence of any commercial or financial relationships that could be construed as a potential conflict of interest.
